# Aging and calorie restriction regulate the expression of miR-125a-5p and its target genes *Stat3*, *Casp2* and *Stard13*

**DOI:** 10.18632/aging.101270

**Published:** 2017-07-31

**Authors:** Kuldeep Makwana, Sonal Arvind Patel, Nikkhil Velingkaar, Jey Sabith Ebron, Girish C. Shukla, Roman V. Kondratov

**Affiliations:** ^1^ Center for Gene Regulation in Health and Disease and Department of Biological, Geological, and Environmental Sciences, Cleveland State University, Cleveland, OH 44115, USA

**Keywords:** MicroRNA, transcription, calorie restriction, aging, longevity

## Abstract

Calorie restriction (CR) is a dietary intervention known to delay aging. In order, to understand molecular mechanisms of CR, we analyzed the expression of 983 MicroRNAs (miRNAs) in the liver of female mice after 2 years of 30% CR using micro-array. 16 miRNAs demonstrated significant changes in their expression upon CR in comparison with age-matched control. mmu-miR-125a-5p (miR-125a-5p) was significantly upregulated upon CR, and in agreement with this, the expression of mRNAs for its three predicted target genes: *Stat3, Casp2, and Stard13* was significantly downregulated in the liver of CR animals. The expression of precursor miRNA for miR-125a-5p was also upregulated upon CR, which suggests its regulation at the level of transcription. Upon aging miR-125a-5p expression was downregulated while the expression of its target genes was upregulated. Thus, CR prevented age-associated changes in the expression of miR-125a-5p and its targets. We propose that miR-125a-5p dependent downregulation of *Stat3, Casp2, and Stard13* contributes to the calorie restriction-mediated delay of aging.

## INTRODUCTION

Aging, a multifactorial process, is accompanied by the decay of many physiological systems and development of various age-associated pathologies such as cancer, osteoporosis, cardiovascular, and metabolic diseases [1–3]. The human population in developed countries in the world is aged, about 15% of the population in the USA will be older than 65 by the year 2020 [4]. As a consequence of aging, the number of age-associated diseases grows, which leads to increased cost of health care [5]. Therefore, the delay of aging and the increase of health span are important tasks of modern biomedical sciences [6]. Calorie restriction (reduction of calorie intake without malnutrition) is a dietary paradigm, which is known to delay aging and reduce the risk of various age-related pathologies [7,8]. Beneficial effects of CR have been reported in various organisms from unicellular to mammals [9,10], yet the mechanism of CR is not well deciphered. Increased resistance to stress including oxidative stress, reduced metabolic rate, changes in insulin/IGF/TOR, and sirtuin signaling pathways have been reported and proposed as potential molecular mechanisms of CR [11–14]. Calorie restriction induced changes in gene expression were also proposed as a contributing factor [15,16]. CR can affect gene expression through multiple mechanisms, such as changes in chromatin modifications, mRNA transcription processing, and mRNA translation. Control of the expression of regulatory RNAs such as miRNAs is an important mechanism of gene expression regulation [17–21].

MicroRNAs (miRNAs) are ∼22nt long RNA molecules, which incorporate into the RNA-induced silencing complex and regulate gene expression through sequence-specific interaction with protein-encoding mRNA transcripts. miRNAs induce either mRNA degradation or translational repression [22]. One miRNA may regulate translation of several different transcripts, and the same mRNA can be regulated by several different miRNAs, thus linking many cellular pathways inside the cell [23]. miRNAs are implicated in regulating the process of aging [20,24] expression of some miRNAs is altered in an age-associated manner and can be reversed by calorie restriction [9,19,20,22].

In this study, we investigated the changes in the expression of miRNAs in the liver of mice subjected to a long-term (two years) calorie restriction. miRNA microarray analysis revealed 16 miRNAs with significant changes in their expression pattern upon CR. One of the miRNA, mmu-miR-125a-5p (miR-125a-5p), and its target genes such as *Stat3, Casp2, and Stard13* demonstrated age-dependent changes, which were ameliorated by CR. Importantly, identified targets have been connected with aging-related signaling pathways. STAT3 is a transcriptional factor that plays a role in the insulin resistance [25], and Casp2 is a proteolytic enzyme, whose activity is upregulated upon aging [26].

## RESULTS

### CR-induced changes in the liver MicroRNA expression

In order to identify miRNAs affected by CR in the liver of mice, we performed the microarray analysis of miRNAs expression in age-matched female animals fed ad libitum (AL) or 30% CR for two years. miRNAs are known to have daily differential expression pattern [27,28]. Therefore, analysis of miRNA's expression performed at one random time of the day might be misleading. To analyze the possible daily differential expression of miRNAs across the day, we collected liver tissues at six different time points across the day (every four hours) for each group and pooled the samples together for analysis. A microarray containing 983 miRNAs on aμParaflo®Microfluidic Biochip Technology Probe was used for the miRNAs microarray analysis. The hybridization signal intensity for 779 miRNAs was below the threshold level. Therefore they were not considered for further analysis. For the remaining 204 miRNAs, the levels of daily average expression and results of statistical analysis are presented in Supplementary Table S1. Among these, CR significantly affected the expressions of 16 miRNAs: 5 miRNAs were down-regulated and 11 were upregulated by CR (see Table 1).

**Table 1:** MicroRNAs, which expression was affected by 30% CR in the liver

Sr.No.	miRNAs	Fold change	p Value	Accession no.
1	mmu-miR-466c-5p	1.6	0.05	MIMAT0004877
2	mmu-miR-145b	1.8	0.02	MIMAT0025105
3	mmu-miR-466m-5p, mmu-miR-669m-5p	1.4	0.04	MIMAT0014882 MIMAT0017346
4	mmu-miR-6240	1.4	0.05	MIMAT0024861
5	mmu-miR-6931-5p	2.1	0.02	MIMAT0027762
6	mmu-miR-125a-5p	1.8	0.003	MIMAT0000135
7	mmu-miR-455-3p	1.6	0.02	MIMAT0003742
8	mmu-miR-139-5p	1.5	0.04	MIMAT0000656
9	mmu-miR-466d-3p	1.6	0.04	MIMAT0004931
10	mmu-miR-5130	2.7	0.04	MIMAT0020641
11	mmu-miR-669h-3p	1.9	0.04	MIMAT0005842
12	mmu-miR-6239	−1.3	0.04	MIMAT0024860
13	mmu-miR-132-3p	−4.7	0.01	MIMAT0000144
14	mmu-miR-6970-3p	−42.9	0.0008	MIMAT0027843
15	mmu-miR-7056-5p	−1.3	0.04	MIMAT0028016
16	mmu-miR-212-3p	−8.1	0.008	MIMAT0000659

**Figure 1:**
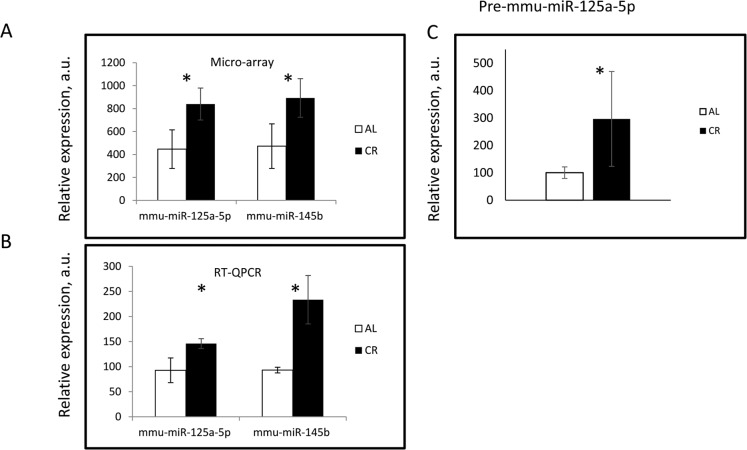
30% CR affected the MicroRNA expression in mouse liver The expression of indicated miRNAs was assayed using (**A**) microarray hybridization and (**B**) Taqman PCR assay in the liver of AL (Open bars) and CR mice (Black bars). * - statistically significant difference (p < 0.05).

To secondary validate the microarray data, we used TaqMan MicroRNA assay to determine miRNAs expression. We selected two miRNAs: mmu-miR-125a-5p, and mmu-miR-145b (miR-145b) which were found to be significantly affected by CR (Figure 1A). Results of the qPCR analysis are presented in (Figure 1B). The expressions of mature miR-125a-5p and miR-145b were significantly higher in the CR group similar to the microarray data (Figure 1 A & B).

miR-125a-5p is well conserved in mouse and human (see Supplementary Table S3). It is implicated in the development of several pathologies and has a role to play in various tissues upon aging [29–37], We decided to further study the regulation of its expression. We checked for the expression of precursor transcript of miR-125a-5p. Gene mir-125a is located on the mouse chromosome 17 spanning from 17,830.812 to 17,830, 879. This gene encodes for 68 nucleotides long pre-cursor (pre-miR-125a-5p), which further upon Post-transcriptional processing generates miR-125a-5p from its 5′ end of the precursor stem. The expression of the precursor was found to be significantly higher in CR samples, shown in (Figure 1C). Thus, both mature MicroRNA and its precursor were upregulated by CR which suggests that the regulation might be controlled at the level of transcription.

### CR affected the expression of Mmu-miR-125a-5p target genes

**Figure 2:**
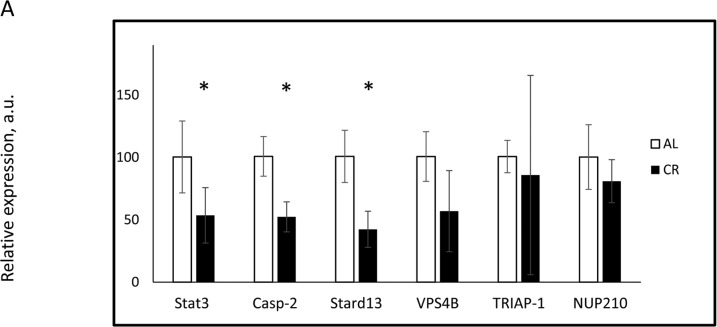
CR affected the expression of miR-125a-5p targeted genes in the mouse liver The expression of indicated mRNAs was assayed in the livers of mice on AL (open bars) or CR (black bars) diets. * - statistically significant difference (p < 0.05).

To study the possible physiological consequence of changes in miR-125a-5p expression, we analyzed the effect of CR on its downstream targets. *Stat3* and *Casp2* were previously reported as targets of miR-125a-5p [32,38]; using bioinformatics we predicted 4 additional targets: *Nup210, Stard13, Triap1*, and *Vps4b*. Details of the seeding sequence and target sites on the 3′UTR of identified targets are presented in Supplementary Table S2. Three targets: *Stat3, Casp2,* and *Stard13* demonstrated significant downregulation at the mRNA level in CR samples compared with AL (Figure 2). These results were in inverse correlation with the data on the upregulation of miR-125a-5p in our study. The expression of *Nup210, Triap1,* and *Vps4b* genes did not show any statistically significant changes upon CR. Thus, CR induced expression of miRNA caused appropriate changes in the expression of some of its target genes suggesting the potential physiological significance of the regulation of miR-125a-5p expression in response to CR.

### CR ameliorated age-induced changes in the expression of miR-125a-5p and its target genes

**Figure 3:**
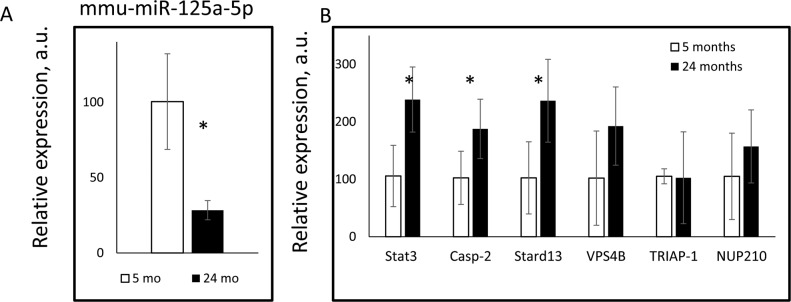
Age dependent changes in the expression of mmu-miR-125a-5p and its target genes The expressions of (**A**) mmu-miR-125a-5p, and (**B**) indicated miR-125a-5p target genes were assayed in the livers of 5 months old (open bars) or 24 month old (black bars) mice. * - statistically significant difference (p < 0.05).

If miR-125a-5p is relevant to aging, its expression might be affected by age. To directly investigate it we compared the expression of miR-125a-5p and its target genes in the liver of young and old mice. As it is shown in Figure 3A, the expression of mature miR-125a-5p was significantly down-regulated with age. These findings were in agreement with previously reported age-dependent downregulation of mature miR-125a-5p expression in the adipose tissues [18]. In agreement with the reduced expression of miR-125a-5p in the liver of aged mice, the expression of *Stat3, Casp2* and *Stard13* was significantly upregulated upon aging (Figure 3B). Thus, aging in mice was accompanied by downregulation of miR-125a-5p expression and by upregulation of the expression of its targets genes such as *Stat3, Casp2,* and *Stard13*. The long-term 30% CR ameliorated age-induced changes in miRNA expression of *Stat3, Casp2, and Stard13*.

**Figure 4:**
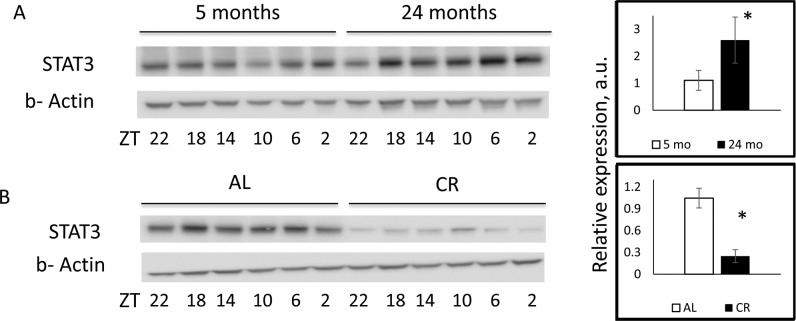
CR reversed age induced upregulation of STAT3 protein expression Representative western blotting and quantification of STAT3 protein expression in the livers of (**A**) 5 month old (open bars) or 24 months old (black bars) mice. (**B**) Mice on AL (open bars) or CR (black bars) mice. * - statistically significant difference (p < 0.05). Western blot images displayed in the figure were not subjected to any manipulation.

To investigate if changes in mRNA level lead to changes at the protein level, we assayed STAT3 protein expression in the liver of young, old, and CR mice. STAT3 expression was upregulated with age in agreement with the upregulation of its mRNA and the downregulation of miR-125a-5p expression (Figure 4A). As it was expected, CR caused a significant reduction in the expression of STAT3 protein in comparison with AL group (Figure 4B). The down-regulation of STAT3 expression at the protein level (80% reduction) was stronger than the downregulation at mRNA level (50% reduction), suggesting that the regulation of the STAT3 expression might occur at both transcript stability and translation levels which are common mechanisms for expression inhibition by miRNAs [39–45].

### Stard13 is a direct target of miR-125a-5p

**Figure 5:**
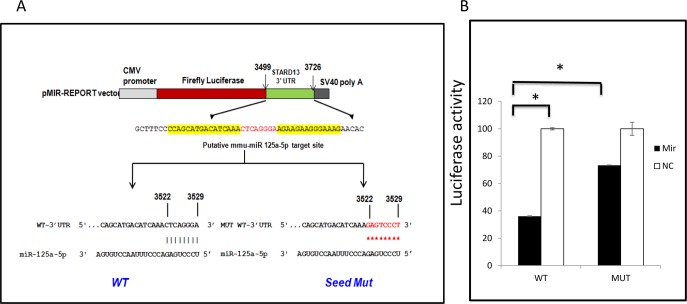
mIR125a-5p targets 3′UTR of *Stard13* (**A**) luciferase reporter construct containing 228 nucleotide sequence from *Stard13* 3′ UTR with either WT or mutant (MUT) miR125a-5p target sites. (**B**) CHO-K1 cells were cotransfected with the constructs presented in figure 5(A) and with mmu-miR 125a-5p mimic or Negative control (NC) as indicated.

Among three targets affected by CR, *Stat3* and *Casp2* are direct targets of miR-125a-5p [32,38]. Therefore, we decided to test whether the reduction in the expression of *Stard13* upon CR is due to the interaction of mmu-miR-125a-5p with the predicted binding on the 3′UTR of *Stard13*. A region of 3′UTR of *Stard13* containing WT or MUT target site was cloned in the firefly luciferase reporter vector (Figure 5A). In the MUT construct, eight nucleotides in the predicted seed region target site of miR-125a-5p on the 3′UTR of *Stard13* were mutated to disrupt the binding of miR-125a-5p. As shown in Figure 5B, miR-125a-5p mimic reduced the luciferase activity of WT construct by about 60% when compared to cells expressing WT 3′UTR of *Stard13* with co-expression of NC mimic. Whereas, there was no change observed in the luciferase activity for the cells expressing MUT construct either with miR-125a-5p mimic or NC mimic. The repression of firefly luciferase activity as observed in the WT cells co-expressing miR-125a-5p mimic was restored by 50% when the miRNA-mRNA interactions were destabilized in the cells co-expressing MUT construct and miR-125a-5p mimic. This experiment concluded that *Stard13* is a direct target *of* miR-125a-5p, therefore, explains the downregulation of *Stard13* expression in animals on CR.

### DISCUSSION

miRNAs are the class of regulatory RNAs that are involved in the control of gene expression. miRNAs are implicated in the regulation of many physiological processes [36,38] including aging and several of them have been reported to be affected by age and their expression is changed in response to CR [17], the feeding paradigm known to delay aging. In this study, we have analyzed the expression of miRNAs in the liver of mice who were subject to 2 years 30% CR. Several of the analyzed miRNAs have been previously reported in connection with aging and CR. miR-125a and miR-145 expression declines upon aging in the mouse subcutaneous adipose tissue and CR restore their levels [18]. We have observed similar changes in the expression of these miRNAs in the liver, hence it is reasonable to predict that they can be regulated by aging and CR in different tissues. In agreement with our data, the downregulation of miR-212-3p by CR in the serum of long-lived B6C3F1 mice have been observed [19], however, we did not observe any significant difference in the expression of other 12 miRNAs which were common for both studies [19]. There are many reasons for the discrepancy; including tissue specificity (liver versus serum), time of feeding, time of tissue collection, and mouse strain all as contributing factors. The expression of miR-93, miR-214, and miR-669c [20-21] in the mouse liver and miR-93 and miR-34a in the rat liver are significantly increased with age [20,46]. In our study, we did not detect any significant effect of CR on miR-214 or miR-669c expression, suggesting that the expression of these two miRNAs is affected by aging but not by CR. The expression of miR-93 and miR-34a were below the detection threshold in our study. The increased expression of mml-miR-451 and mml-miR-144 in the skeletal muscle of old rhesus monkey is reversed by CR [17], but in our study, we did not see any significant effect of CR on the expression of mmu-miR-451, whereas expression of mmu-miR-144 was below the detection threshold. Thus, our and previous study suggest that miRNAs expression is affected by age and diets, but effects are tissue and species specific.

The upregulation of miR-125a-5p expression was also observed at the level of precursor RNA (Figure 1C). EGFR signaling pathway has been reported to be implicated in the regulation of miR-125a-5p expression [35], but to the best of our knowledge, the regulation of miR-125a-5p transcription or post-transcriptional processing has not been studied.

miR-125a-5p is known to be involved in the regulation of cell death [34,47], inflammatory response and lipid uptake in monocytes/macrophages [31]. miR-125a-5p suppresses tumorigenesis and inhibits metastasis [33,35], which is in agreement with known antitumor effects of CR. Since miRNAs target the expression of multiple genes, we performed bioinformatics analysis, identified several candidates and analyzed the effect of CR on their expression. The expression of *Stat3, Stard13,* and *Casp2* was significantly downregulated by CR at mRNA level, and STAT3 was also downregulated at the protein level, which is inversely correlated with the upregulation of miR-125a-5p. In agreement with age-dependent changes in the expression of miR-125a-5p (Figure 3A) the expression of its targets: *Stat3, Casp2, and Stard13* was increased with age (Figure 3B) and reversed by CR (Figure 2). Therefore, most likely, the regulation of these genes by age and CR occurs in a miRNA-dependent manner. Importantly, *Stat3 and Casp2* previously reported as targets for miR-125a-5p [32,38].

Effect of aging and CR on STAT3 expression and activity has been previously reported [48,49]. In one study, the increased STAT3 activation (nuclear translocation) in response to growth hormone stimulation has been observed in the liver slices from CR animals compare with slices from AL group, while no significant effect of aging or CR at the total level of STAT3 protein has been detected [48]. In another study, the increase in leptin-stimulated phosphorylation of STAT3 in the hypothalamus was observed in CR group and again no change in the total STAT3 protein level was observed [49]. In our study, we have demonstrated age-dependent upregulation of total STAT3 (Figure 4A) and amelioration of this upregulation upon long-term 30% CR (Figure 3). Together all these results suggest that the effect of diet and age on STAT3 is complicated and might be tissue and treatment specific.

We performed Kyoto Encyclopedia of Genes and Genomes (KEGG) pathway database analysis and identified several pathways relevant to aging, which might be affected by changes in STAT3 level. Chronic activation of STAT3 has been reported to play a part in the development of insulin resistance by increasing the levels of SOCS1 and SOCS3 proteins. SOCS1 and SOCS3, in turn, cause the degradation of insulin receptor substrate IRS-1 and IRS-2, thus, impairing insulin signaling [50–52]. The insulin resistance is one of the manifestations of aging [25] and our data on changes in STAT3 expression with age are in good agreement with age-induced insulin resistance. The prevention of STAT3 induction by CR might contribute to well-known improvement in insulin sensitivity by CR [53,54]. Another potential role of STAT3 in aging revealed by KEGG pathway analysis is the regulation of the RAGE signaling. RAGE is a type I cell surface receptor for advanced glycation end products (AGE) which might contribute to deregulation of the inflammatory response with age [55,56].

Another target of miR-125a-5p, whose expression was affected by CR, is *Casp2*. CASP2 is a cysteine protease that performs site-specific proteolysis of many intracellular proteins and it is linked to apoptosis [57,58]. The activity of several caspases, including CASP2, has been found to be increased in multiple organs of rats upon aging [26]. It was proposed that the increased caspase activity with age is due to the oxidative stress, and high level of apoptosis in the liver of old MnSOD^+/−^mice supports this speculation [57–59]. CR inhibits caspase activity which might be due to the reduced oxidative stress [11,60,61] or due to the preventive effect on the age-dependent upregulation of *casp2* as we demonstrated here.

Many age-related pathologies have been associated with the deregulated apoptosis, indeed, failure in the efficient removal of autoreactive or malignant cells lead to the development of auto-immune diseases and cancers. The excessive removal of irreplaceable cells results in organ atrophy and dysfunction, as seen in many aging-associated pathologies like neurodegenerative diseases, kidney diseases, cardiovascular dysfunction and intestinal disorders [61]. Reduced number of hepatocytes and reduction in liver size have been reported upon aging [57,62]. We speculate that via miR-125a-5p mediated manner CR maintains an optimal basal level of CASP2 protein and, thus, maintain basal CASP2 activity to maintain the homeostasis of the liver tissue by decreasing the aging-induced apoptosis.

The third target of miR-125a-5p, whose expression was affected by aging and CR was *Stard13*, also known as *DLC2*. Our study has provided data that suggests *Stard13* to be a direct target of miR-125a-5p (Figure 5A & B). STARD13 is a Rho GTPase-activating protein, which has been suggested to regulate actin cytoskeleton by inactivating Rho GTPases. It was proposed that STARD13 has tumor suppressor functions, which is supported by cell culture-based experiments but not by *in vivo* studies [63,64]. CR has known anti-tumor activity in mammals, hence downregulation of the expression of a potential tumor suppressor gene is counterintuitive, however, *Stard13* might have some other functions, which cannot be ruled out.

There are questions that need to be answered in future studies. What are the molecular mechanisms of the regulation of miRNAs expression by age and by CR? Will the same set of miRNAs be affected in different tissues upon CR? Is it possible to achieve the metabolic effects of CR by modulating the expression of certain miRNAs? Will the same mechanisms also be functional in primates taking into account that some of identified miRNAs are conserved between rodents and humans? Our results support the importance of miRNA regulation and their pro- and anti-aging activities and warrant further study of identified miRNAs and their targets to understand the mechanism of aging and calorie restriction.

## METHODS

### Ethics statement

The care and use of mice were carried out in accordance with the guidelines of the Institutional Animal Care and Use Committee (IACUC) of the Cleveland State University. Approval from the Institutional Animal Care and Use Committee (IACUC) of Cleveland State University (Protocol No. 21124-KON-S) was obtained for all the performed animal studies.

### Experimental animals

Wild-type mice of C57B6J background were used for the study. Animals were maintained on the 12:12 light: dark cycles with lights turned off and on at 7 am and 7 pm respectively. Animals were fed 18% protein rodent diet (Harlan). The ad libitum (AL) group, had unlimited access to the food all the time. Calorie restriction was implemented gradually. At the age of 3 months, animals were fed 10% calorie restriction diet for the first week i.e. the animals were fed 90% of their total daily food consumption. The second week, animals were maintained on 20% calorie restriction, and after that on 30% calorie restriction diet for two years. The CR group received food as a single meal two hours after the lights were turned off i.e.at ZT14 (Zeitgeber). AL and CR group had unlimited access to water. All the experiments were performed on female mice. At the time of tissue collection, animals were 24 months old.

### Cell culture, transfection, and luciferase assay

CHO-K1 cells were maintained in Dulbecco's Modified Eagle's Medium (DMEM: 5% FBS, 2 mM L-glutamine, 1 mM L-proline, 10 mM HEPES) in humidified 5% CO_2_ atmosphere at 37°C. For Wild-type (WT) reporter construct, 228bp of 3′UTR of *Stard13* gene spanning putative site for mmu-miR-125a-5p was cloned downstream of firefly luciferase coding region in pMIR-REPORT vector (Ambion, Austin, TX). To generate the mutated construct (MUT), site-directed mutagenesis of the putative target was carried out in the WT-3′ UTR construct. Primers used for the cloning and mutagenesis are listed in the Table 2. CHO-K1 cells were plated in 24 well plate one day before the transfection. Cells were co-transfected with 100ng of WT-3′UTR or MUT-3′UTR firefly luciferase reporter construct, 0.5 ng of renilla luciferase reporter plasmid (Promega, Madison, WI) and with either miR-125a-5p mimic(GE lifesciences) or Negative control (NC) mimic (Ambion catalog# 4464058) in duplicates. Cell lysates were collected 48hours after transfection and assayed for luciferase activity using Dual-Luciferase Reporter Assay System (Promega) and Victor 3 Multilabel Counter 1420 (PerkinElmer). Renilla luciferase activity was used to normalize the firefly luciferase activity.

**Table 2:** Primers used to clone wild type and mutated 3′UTR of Stard13 mRNA

STARD13 3′UTR	5′-3′
Forward	ATGCACTAGTGCTTTCCCCAGCATGACATC
Reverse	ATGCAAGCTTTAGGCAACGCGGAAGAAATA
Mutant primer	CCAGCATGACATCAAAGAGTCCCTAGAAGAAGGGAAAG

### RNA isolation and mRNA expression analysis

To study the gene expression, liver tissues were collected from six animals, each from AL and CR group. Tissues were collected at six different time points across the day (ZT2, ZT6, ZT10, ZT14, ZT18, and ZT22). Liver tissues collected, were snap frozen on dry ice and stored at -80°C. TriZol reagent was used to isolate the total RNA from the liver tissues according to the manufacturer's protocol (Invitrogen, Carlsbad, CA), and concentration was determined using Nanodrop (Thermo Scientific Nanodrop 2000). RNA expression analysis was performed using real-time RT-PCR with Universal Syber Green mix (BioRad, Hercules, CA) as previously described [65]. Sequences of the primers used for the analysis of expression are listed in the Table 3).

**Table 3:** Primers used for the analysis of mRNA expression for miR-125a-5p target genes

Gene	Forward (5′-3′)	Reverse (5′-3′)
Casp2	ACCGTTGAGCTGTGACTATG	GTTCCGTAGCATCTGTGGATAG
Stat3	GTCTGTAGAGCCATACACCAAG	GGTAGAGGTAGACAAGTGGAGA
Nup210	CTCACTCTCATGCCTGTCTATG	GGATTACGGTGACTGGATACTG
Vps4b	CAACGGAAGCAAACAACTCAA	CTCTCTGGCAAGCTGGAATAA
Stard13	CAGGGAAGAAGAAGGGAAAGAA	GGCAACGCGGAAGAAATAAC
Triap1	GGATTAAGGATGGCAGCATTTG	GAGCTCCAAAGTTGGTGTTTATG

### TaqMan assays for analysis of the mature MicroRNA expression

Taqman assays (ThermoFisher Scientific) were used to validate the results obtained from MicroRNA micro-array analysis. Reverse transcription was carried out using reverse transcription kit (ThermoFisher Scientific #4366596) as per the manufacturer's protocol. Total RNA used per reaction for the reverse transcription step was 100ng. Determination of miRNA expression of miR-125a-5p and miR-145b was done by using TaqMan micro RNA assay (ThermoFisher Scientific Catalog # 4427975), and TaqMan Fast Universal PCR master mix 2x (ThermoFisher Scientific Catalog # 4352042) as per the manufacturer's instructions. The detection system used was CFX96 qPCR (BioRad). The reactions for the gene of interest were carried out in triplicates, while normalizing control was run in duplicates. Validation was done using the same RNA samples, which were used for micro array analysis. The comparative delta-Ct method was used to quantify the results. The normalizing control used was snoRNA202 (ThermoFisher Scientific #4427975).

### MicroRNA microarray analysis

To assay miRNAs expression profiles, six samples from each AL and CR group were subjected to microarray analysis. Microarray analysis and the data generation was all carried out by LC SCIENCES (Houston, Texas) using μParaflo®Microfluidic Biochip Technology. Probe content for all the mature miRNAs was according to the updated version of Sanger miRBase database. All the miRNAs selected, were having a hybridization signal intensity value of 400 or more for an at least one-time point in either of the group.

### Immunoblot analysis

Liver tissue samples were immediately frozen on dry ice upon harvesting and stored at -80C before the analysis. Protein extracts were prepared by the lysis of frozen liver tissues in the Cell signaling lysis buffer with the Protease/Phosphatase Inhibitor cocktail (Cell Signaling Technology, Beverly, MA, USA). Bradford protein assay kit was used to determine the protein concentration using spectrophotometer. Lysates were stored at -80°C. 30ug protein was loaded per well on 4-12% bis-tris gels. Protein was transferred onto the PVDF membrane at 110mA for 70 minutes. The membrane was stained with Ponceau stain to check for the equal loading of proteins. Primary antibody against STAT3 (D1A5) was purchased from Cell Signaling. Staining of the same membranes for beta-actin (SIGMA-ALDRICH #A5441) was used for normalization. All the samples were run on the same gel. Images were obtained with Odyssey FC imaging system (LI-COR, Lincoln, NE), and quantification was performed with Image Studio Lite version 5.2 software.

### MicroRNA target site prediction and pathway analysis

Following web resources: Targetscan, miRecords, MicroRNA.org, and mirdb were used to predict targets of individual miRNAs. RNAhybrid - BiBiServ and Targetscan were used to predict the putative target site for miR-125a-5p in the 3′UTR region of the target. KEGG pathway database analysis was done for the targets of miR-125a-5p.

### Statistical analysis

For quantitative data, liver tissue samples collected at six different time points across the day for AL and CR groups were run individually. Results are presented as average values plus/minus standard deviation. IBM SPSS Statistics20 and GraphPad Prism Version 5.04software packages were used for statistical analysis. To assay the effect of the diet, unpaired two-tailed T-test was performed. P<0.05 was considered as a statistically significant difference.

## SUPPLEMENTARY MATERIAL TABLES



## Abbreviations

miRNAs: MicroRNA
CR: Calorie Restriction
AL: Adlibitum
miR-125a-5p: mmu-miR-125a-5p
miR-145b: mmu-miR-145b
Casp2: Caspase2
WT: Wild Type
MUT: Mutant
NC: Negative Control
