# Supplementary material for: Aging and calorie restriction regulate the expression of miR-125a-5p and its target genes Stat3, Casp2 and Stard13
Source: Aging (Albany NY). 2017 Jul 31;9(7):1825–37. doi: 10.18632/aging.101270 (PMC5559175; doi:10.18632/aging.101270)
Supplement: Supplementary file 1 [file aging-09-1825-s001.pdf]

SUPPLEMENTARY MATERIAL

Supplementary Table S1. The effect of 30% CR on miRNA expression in the liver.

	Group 1		Group 2		
	Cr		Ad		
Reporter Name	Mean	StDev	Mean	StDev	T-test(p-Value)
mmu-miR-3960	7,214	3,720	5,893	1,870	0.345729
mmu-miR-7028-5p	10,850	5,504	11,422	12,795	0.707084
mmu-miR-133b-5p	29,166	4,790	27,453	17,687	0.996587
mmu-let-7k	347	174	359	360	0.770206
mmu-miR-3962	317	180	368	322	0.465436
mmu-miR-3620-5p	2,201	1,202	2,010	944	0.806528
mmu-miR-669c-5p	1,567	488	1,633	403	0.863513
mmu-miR-2861	2,335	1,637	1,198	426	0.039967
mmu-miR-669f-5p	939	270	934	236	0.810106
mmu-miR-6238	615	747	2,960	4,161	0.048597
mmu-miR-341-5p	56,348	8,592	47,700	12,934	0.012713
mmu-miR-101b-3p	1,044	446	1,213	426	0.326458
mmu-miR-100-5p	319	96	438	247	0.091844
mmu-miR-143-3p	1,202	628	911	121	0.131428
mmu-miR-27a-3p	1,034	309	1,105	321	0.494768
mmu-miR-15a-5p	968	470	1,402	576	0.036146
mmu-miR-669o-5p	396	150	354	105	0.454633
mmu-miR-195a-5p	496	315	490	155	0.984438
mmu-miR-6937-5p	4,972	2,040	4,313	1,551	0.506479
mmu-miR-669l-5p	419	149	380	111	0.502784
mmu-miR-669n	1,609	472	1,693	423	0.773574
mmu-miR-466g	1,736	510	1,625	366	0.559514
mmu-miR-7047-5p	3,664	1,589	3,539	1,266	0.978717
mmu-miR-466c-5p	599	206	365	139	0.006519
mmu-miR-24-3p	3,017	1,225	2,871	309	0.641511
mmu-miR-194-5p	6,894	1,425	7,668	1,980	0.318193
mmu-miR-149-3p	3,665	1,945	3,240	1,146	0.656297
mmu-miR-25-3p	512	235	469	85	0.506317
mmu-miR-3099-3p	564	144	561	125	0.918921
mmu-let-7j	511	131	557	251	0.504248
mmu-miR-466j	409	205	574	291	0.235113
mmu-miR-101c	274	173	303	292	0.601243
mmu-miR-2137	1,320	732	915	382	0.127494
mmu-miR-148a-3p	2,704	672	3,054	912	0.241186
mmu-miR-5100	1,232	940	700	95	0.077305
mmu-miR-762	5,644	1,968	5,078	2,189	0.629904
mmu-let-7e-5p	273	151	234	252	0.811858
mmu-miR-30e-5p	1,994	950	2,014	604	0.860057
mmu-miR-466h-5p	265	185	504	262	0.031873
mmu-miR-3473b	875	534	465	216	0.037754
mmu-miR-669k-5p	387	154	383	81	0.86831
mmu-miR-466h-3p	3,202	708	3,282	558	0.878888
mmu-miR-3095-3p	877	192	826	189	0.531237
mmu-miR-466i-5p	3,884	832	4,156	1,033	0.674214
mmu-miR-30a-5p	3,669	1,104	3,600	731	0.907727
mmu-miR-145b	893	219	473	288	0.001227
mmu-miR-466m-5p	926	269	632	134	0.003522

mmu-miR-29b-3p	894	666	1,157	637	0.264488
mmu-miR-151-5p	334	135	334	124	0.986799
mmu-miR-6970-5p	512	314	399	154	0.35769
mmu-miR-27b-3p	1,737	431	1,888	355	0.45367
mmu-miR-1934-3p	399	266	277	154	0.244947
mmu-miR-221-3p	786	251	735	162	0.519058
mmu-miR-3072-5p	385	123	455	167	0.247301
mmu-miR-669c-3p	2,285	623	2,092	361	0.432303
mmu-miR-3077-5p	392	327	221	104	0.125798
mmu-miR-669f-3p	1,807	479	1,663	241	0.420768
mmu-miR-6240	502	107	345	128	0.00734
mmu-miR-3970	68	41	223	257	0.034218
mmu-miR-5126	8,401	3,751	6,718	1,756	0.206182
mmu-miR-7683-3p	136	135	318	369	0.096302
mmu-miR-3535	395	204	254	123	0.048215
mmu-let-7g-5p	3,091	535	2,883	715	0.330805
mmu-miR-6239	14,009	3,189	18,268	3,055	0.00601
mmu-miR-6931-5p	801	377	366	108	0.001608
mmu-miR-378c	329	127	355	83	0.772099
mmu-miR-6385	425	140	443	89	0.783923
mmu-miR-485-3p	529	127	572	231	0.861091
mmu-miR-101a-3p	1,160	558	1,346	555	0.357532
mmu-miR-378a-3p	446	149	514	129	0.396116
mmu-miR-6366	277	138	266	75	0.865699
mmu-miR-26b-5p	2,069	582	1,718	405	0.111709
mmu-miR-3473f	488	363	199	76	0.021179
mmu-let-7f-5p	3,119	892	2,538	680	0.092733
mmu-miR-7045-5p	1,231	502	1,132	268	0.638869
mmu-miR-23b-3p	2,288	634	2,160	503	0.508675
mmu-miR-705	870	350	736	199	0.344456
mmu-miR-3082-5p	1,533	430	1,541	380	0.937836
mmu-miR-1892	557	337	521	256	0.839428
mmu-miR-3963	1,215	695	1,138	366	0.861965
mmu-miR-6908-5p	272	110	372	124	0.081446
mmu-miR-22-3p	7,143	1,381	7,467	1,743	0.688749
mmu-miR-5099	2,857	903	2,234	497	0.056274
mmu-miR-20b-5p	193	101	289	171	0.072652
mmu-miR-466q	1,750	494	1,560	372	0.346314
mmu-miR-7011-5p	340	117	435	96	0.039827
mmu-miR-6965-3p	689	371	2,111	2,373	0.037218
mmu-miR-423-5p	319	59	353	188	0.492977
mmu-miR-107-3p	1,048	372	1,331	287	0.08274
mmu-miR-329-3p	34	20	144	316	0.210463
mmu-miR-7221-3p	1,527	853	1,031	348	0.106025
mmu-miR-30c-5p	5,378	1,120	5,293	1,404	0.797422
mmu-miR-466f	526	197	537	212	0.779986
mmu-miR-145a-5p	1,726	554	1,417	209	0.087719
mmu-let-7d-5p	2,497	735	2,091	837	0.265377
mmu-miR-20a-5p	819	292	805	182	0.88494
mmu-miR-17-5p	558	209	616	187	0.481723
mmu-miR-363-5p	132	84	409	327	0.008187
mmu-miR-21a-5p	5,170	2,064	4,156	1,694	0.148317
mmu-miR-466f-3p	3,338	638	3,353	456	0.988181
mmu-miR-5130	332	215	120	48	0.004882
mmu-miR-7082-5p	2,063	640	2,862	1,106	0.0436

mmu-miR-378b	350	147	371	90	0.871018
mmu-miR-466i-3p	2,524	582	2,427	388	0.626978
mmu-miR-30d-5p	2,078	619	1,832	851	0.539806
mmu-miR-466f-5p	483	153	373	129	0.024391
mmu-miR-5119	277	189	218	118	0.451497
mmu-miR-6929-3p	326	72	368	110	0.387556
mmu-miR-6981-5p	941	263	1,165	229	0.044817
mmu-miR-130a-3p	967	286	1,011	223	0.777342
mmu-miR-93-5p	434	182	458	65	0.70582
mmu-let-7i-5p	845	236	864	270	0.89157
mmu-miR-7658-5p	248	191	118	40	0.039922
mmu-miR-1187	1,765	391	2,275	725	0.084365
mmu-miR-5107-5p	429	174	648	210	0.008225
mmu-miR-346-3p	626	302	468	183	0.21009
mmu-miR-122-3p	2,205	817	2,301	1,003	0.780025
mmu-miR-669e-5p	154	124	287	151	0.055
mmu-miR-342-3p	160	82	231	153	0.139103
mmu-miR-29c-3p	2,844	1,239	3,114	1,290	0.501129
mmu-miR-3473e	702	435	333	144	0.019497
mmu-miR-152-3p	537	190	553	191	0.830174
mmu-miR-5113	512	135	468	134	0.473942
mmu-miR-22-5p	479	165	506	138	0.764314
mmu-miR-214-3p	546	374	536	92	0.948595
mmu-miR-103-3p	1,064	398	1,298	295	0.180138
mmu-let-7a-5p	2,818	843	2,284	920	0.193003
mmu-miR-802-5p	412	256	386	250	0.935593
mmu-miR-6944-5p	660	359	512	201	0.328479
mmu-miR-181a-5p	419	533	207	24	0.200903
mmu-miR-6896-3p	621	147	679	183	0.487437
mmu-miR-8101	315	160	306	109	0.956405
mmu-miR-494-3p	306	108	607	455	0.020016
mmu-miR-690	12,388	2,556	12,478	1,637	0.865777
mmu-miR-466m-3p	1,589	411	1,530	227	0.683266
mmu-miR-146a-5p	571	222	642	231	0.454848
mmu-miR-6922-5p	492	201	502	116	0.907653
mmu-miR-669a-3p	1,975	540	1,877	276	0.645677
mmu-miR-1895	875	202	1,151	258	0.012891
mmu-miR-30b-5p	3,305	908	3,238	856	0.850504
mmu-miR-672-5p	245	137	441	238	0.041637
mmu-miR-92b-3p	420	175	315	64	0.086432
mmu-miR-203-3p	453	118	519	178	0.39029
mmu-miR-8093	54	60	138	285	0.287563
mmu-miR-3097-5p	377	157	397	231	0.854942
mmu-miR-99a-5p	863	298	887	402	0.779772
mmu-miR-31-5p	749	226	759	158	0.958095
mmu-miR-669e-3p	1,689	440	1,579	195	0.529697
mmu-miR-122-5p	10,790	972	11,909	2,243	0.237239
mmu-miR-1224-5p	419	135	432	91	0.707676
mmu-miR-23a-3p	2,104	569	1,890	487	0.323776
mmu-miR-5112	4,114	2,707	6,888	5,212	0.071404
mmu-miR-7661-3p	280	96	388	91	0.018606
mmu-let-7c-5p	2,337	706	1,949	839	0.276307
mmu-miR-451a	4,801	844	4,358	1,396	0.431628
mmu-miR-32-3p	785	266	719	202	0.528477
mmu-miR-669a-3-3p	739	278	579	153	0.131556

mmu-miR-7239-3p	3,747	4,414	8,162	10,140	0.133449
mmu-miR-15b-5p	412	152	321	82	0.092767
mmu-miR-7027-3p	283	53	296	106	0.764451
mmu-miR-19b-3p	492	262	562	197	0.594425
mmu-miR-26a-5p	5,655	751	5,397	1,012	0.302324
mmu-miR-7081-5p	3,523	2,029	5,329	5,554	0.203969
mmu-miR-125a-5p	840	158	446	194	4.533E-5
mmu-miR-467a-3p	1,210	378	1,079	286	0.412676
mmu-miR-5121	518	205	392	82	0.044916
mmu-miR-7235-3p	594	93	649	180	0.463036
mmu-miR-467f	2,627	602	2,567	465	0.768961
mmu-miR-714	413	245	250	80	0.062626
mmu-let-7b-5p	1,444	466	1,290	624	0.585893
mmu-miR-467e-3p	740	248	593	164	0.143019
mmu-miR-467c-3p	880	293	736	194	0.220199
mmu-miR-574-5p	2,597	552	3,040	938	0.295107
mmu-miR-483-5p	1,085	329	1,260	261	0.182766
mmu-miR-212-3p	32	12	259	171	0.000167
mmu-miR-6348	1,258	426	1,403	400	0.542885
mmu-miR-467g	701	242	512	172	0.053295
mmu-miR-574-3p	1,659	415	1,475	342	0.311189
mmu-miR-126a-3p	4,832	1,128	4,781	914	0.920253
mmu-miR-7016-5p	269	63	363	209	0.112953
mmu-miR-467b-3p	2,685	618	2,764	444	0.805065
mmu-miR-199a-5p	342	295	301	45	0.670773
mmu-miR-468-3p	428	145	357	159	0.113217
mmu-miR-3569-5p	262	75	337	93	0.029548
mmu-miR-467d-3p	2,044	547	2,004	398	0.865427
mmu-miR-192-5p	6,114	1,229	6,908	1,594	0.294265
mmu-miR-7116-5p	285	368	396	290	0.335222
mmu-miR-199a-3p	605	491	625	168	0.888147
mmu-miR-700-3p	259	86	329	184	0.192203
mmu-miR-191-5p	1,121	397	1,162	270	0.812239
mmu-miR-200b-3p	181	161	113	45	0.156052
mmu-miR-1191b-5p	106	131	129	275	0.705775
mmu-miR-328-5p	274	91	319	119	0.214503
mmu-miR-455-3p	371	108	231	71	0.00106
mmu-miR-200a-3p	222	205	145	55	0.210269
mmu-miR-16-5p	4,536	997	5,326	875	0.069498
mmu-miR-709	13,326	2,032	16,085	2,474	0.012694
mmu-miR-8090	53	75	138	334	0.35321
mmu-miR-92a-3p	800	306	670	132	0.170368
mmu-miR-7056-5p	347	99	484	112	0.002219
mmu-miR-139-5p	261	90	166	43	0.004586
mmu-miR-466d-3p	260	111	153	39	0.008479
mmu-miR-6970-3p	47	15	2,013	1,567	0.000219
mmu-miR-132-3p	73	38	345	371	0.014901

Supplementary Table S2. Conserved putative binding sites for mmu-miR-125a-5.

<table>
<tr><th rowspan="2">Gene</th><th rowspan="2">mmu-miR-125a-5p:3'UTR base-pairing</th><th colspan="2">Position on 3'UTR</th></tr>
<tr><th>Start</th><th>End</th></tr>
<tr><td>Stat3</td><td>target 5' C UG CCUGCCUCAGACUAC CCCUCAGCAAAG G 3'
UUACAGGUU AA AGG CUCAGGGA
||||||||| || ||| ||||||||
AGUGUCCAA UU UCC GAGUCCCU
miRNA 3' CA 5'</td><td>969</td><td>1020</td></tr>
<tr><td>Caspase2</td><td>target 5' UUCUGUCUACCCUCCCUCUCAGGGA 3'
|||||||
miR 3' .AGUGUCCAAUUUCCCAGAGUCCCU 5'</td><td>76</td><td>100</td></tr>
<tr><td>Stard13</td><td>target 5' ...CAGCAUGACAUCAAACUCAGGGA 3'
|||||||
miR 3' AGUGUCCAAUUUCCCAGAGUCCCU 5'</td><td>9</td><td>31</td></tr>
<tr><td>Triap1</td><td>target 5' C GCUCG UC ACCUCUUUUUCC U 3'
UCAUGGG G GGG UCUCAGGGA
||||||| | ||| |||||||||
AGUGUCC U CCC AGAGUCCCU
miRNA 3' AA UU 5'</td><td>328</td><td>367</td></tr>
<tr><td>Nup210</td><td>target 5' G U U A A 3'
UCAC GU AG G UCUCAGGGA
|||| || || | |||||||||
AGUG CA UC C AGAGUCCCU
miRNA 3' UC AUU C 5'</td><td>349</td><td>371</td></tr>
<tr><td>Vps4b</td><td>target 5' U A UCUUUAC U 3'
GGU GAA UCUCAGGGA
||| ||| |||||||||
CCA UUU AGAGUCCCU
miRNA 3' AGUGU A CCC 5'</td><td>434</td><td>458</td></tr>
</table>

Supplementary Table S3. The sequence conservation of miR125a-5p between mouse and human.

Specie	MiR-125a-5p sequence
Mouse	5’UCCCUGAGACCCUUUAACCUGUGA 3’
Human	5’UCCCUGAGACCCUUUAACCUGUGA 3’
